# Efficient secretory production of proline/alanine/serine (PAS) biopolymers in *Corynebacterium glutamicum* yielding a monodisperse biological alternative to polyethylene glycol (PEG)

**DOI:** 10.1186/s12934-022-01948-5

**Published:** 2022-10-28

**Authors:** L. Friedrich, Y. Kikuchi, Y. Matsuda, U. Binder, A. Skerra

**Affiliations:** 1 XL-protein GmbH, Lise-Meitner-Strasse 30, 85354 Freising, Germany; 2 Research Institute for Bioscience Products & Fine Chemicals, Ajinomoto Co., Inc., 1-1 Suzuki-cho, Kawasaki-ku, Kawasaki, 210-8681 Japan; 3 Lehrstuhl für Biologische Chemie, Technische Universität München, Emil-Erlenmeyer-Forum 5, 85354 Freising, Germany

**Keywords:** Disordered polypeptide, Hydrophilic polymer, PASylation, PEG, Pro/Ala/Ser

## Abstract

**Background:**

PAS biopolymers are recombinant polypeptides comprising the small uncharged l-amino acids Pro, Ala and/or Ser which resemble the widely used poly-ethylene glycol (PEG) in terms of pronounced hydrophilicity. Likewise, their random chain behaviour in physiological solution results in a strongly expanded hydrodynamic volume. Thus, apart from their use as fusion partner for biopharmaceuticals to achieve prolonged half-life in vivo, PAS biopolymers appear attractive as substitute for PEG—or other poorly degradable chemical polymers—in many areas. As a prerequisite for the wide application of PAS biopolymers at affordable cost, we have established their highly efficient biotechnological production in *Corynebacterium glutamicum* serving as a well characterized bacterial host organism.

**Results:**

Using the CspA signal sequence, we have secreted two representative PAS biopolymers as polypeptides with ~ 600 and ~ 1200 amino acid residues, respectively. Both PAS biopolymers were purified from the culture supernatant by means of a simple downstream process in a truly monodisperse state as evidenced by ESI–MS. Yields after purification were up to ≥ 4 g per liter culture, with potential for further increase by strain optimization as well as fermentation and bioprocess development. Beyond direct application as hydrocolloids or to exploit their rheological properties, such PAS biopolymers are suitable for site-specific chemical conjugation with pharmacologically active molecules via their unique terminal amino or carboxyl groups. To enable the specific activation of the carboxylate, without interference by the free amino group, we generated a blocked N-terminus for the PAS(1200) polypeptide simply by introducing an N-terminal Gln residue which, after processing of the signal peptide, was cyclised to a chemically inert pyroglutamyl group upon acid treatment. The fact that PAS biopolymers are genetically encoded offers further conjugation strategies via incorporation of amino acids with reactive side chains (e.g., Cys, Lys, Glu/Asp) at defined positions.

**Conclusions:**

Our new PAS expression platform using Corynex^®^ technology opens the way to applications of PASylation^®^ technology in multiple areas such as the pharmaceutical industry, cosmetics and food technology.

## Introduction

Poly-ethylene glycol (PEG) is a robust and strongly hydrophilic chemical polymer, available in different length distributions and, optionally, with various functionalized end groups, which has found wide application as reagent in biochemical and biophysical research, as ingredient in cosmetics and food products as well as in medical therapy. In particular, chemical conjugation of recombinant proteins with PEG, also known as PEGylation, has led to multiple clinically approved biopharmaceuticals, mainly with the goal of prolonging their intrinsically short circulation after parenteral administration [1, 2].

However, with regard to therapeutic application, the use of PEG has a number of practical caveats, in particular the high cost of goods for the chemically activated substance in clinical grade and the inherent polydispersity of the chemical polymer, which necessitates elevated effort for quality analysis [3]. Even more importantly, PEG is not degraded by enzymes in the human body, and cases of tissue accumulation and/or PEG vacuole formation were repeatedly described in preclinical studies [4]. Furthermore, while PEGylation was initially developed to achieve shielding of highly immunogenic bacterial enzymes [5], incidences of PEG hypersensitivity have been reported; and this situation may become even more prevalent as a consequence of current world-wide vaccination campaigns with PEG-coated lipid nanoparticles (LNPs) that are used to encapsulate Corona virus antigen mRNA [6].

Recently, structurally disordered and highly soluble polypeptides comprising the small L-amino acids Pro, Ala and/or Ser (PAS) were developed as a biological and biodegradable alternative to PEG [7–9]. Such PAS sequences can be genetically encoded, thus allowing the efficient production of fusion proteins with a variety of biologically or pharmacologically active proteins and peptides including antibody fragments, growth factors, cytokines, adipokines and enzymes [10]. Furthermore, PAS sequences can be employed as linkers between different proteins or functional domains [11–13].

So far, PASylation^®^ technology was mainly applied in preclinical and clinical settings in order to extend the plasma half-life of a therapeutic or diagnostic biological entity [14–21]. Indeed, the strongly expanded hydrodynamic volume of the natively unfolded PAS polypeptide effectively retards kidney filtration and leads to prolongation of the circulation half-life by 1–2 orders of magnitude, depending on the length of the PAS chain (typically, in the range of 100–800 residues). A similar size effect can also be exploited to prolong the ocular half-life of biopharmaceuticals after intravitreal administration [13].

In addition, PASylation can be applied to stabilize and shield the surface of nanoparticles due to the pronounced hydrophilicity and biochemically inert nature of PAS chains. For example, PASylation of nanocages based on human ferritin (FRT) facilitates doxorubicin encapsulation, reduces drug leakage and significantly prolongs their plasma half-life in mice [22]. In fact, PASylation can solve a common problem of nanoparticles, i.e. the association with plasma proteins such as antibodies and complement proteins, which can cause immune clearance and very short circulation [23]. PASylation was also applied for the coating of plasmid DNA lipoplexes, yielding in vitro properties similar to PEGylation [24].

Further to the use in form of fusion proteins, PAS biopolymers have been prepared as isolated substances, for example by site-specific cleavage from a dummy fusion partner after production in *Escherichia coli* [9]. Their detailed biophysical characterization revealed an astonishing similarity between the PAS biopolymers and PEG in equivalent chain lengths—both representing uncharged macromolecules—in terms of conformational random coil behaviour and high solubility not only in aqueous buffer solutions but also in polar organic solvents. On the other hand, PAS biopolymers offer three eminent advantages: (i) enzymatic biodegradability and traceless metabolization if taken up by cells, (ii) low to no immunogenicity in all animal studies performed so far, and (iii) strict monodispersity, which strongly facilitates bioprocess development and drug product analysis, e.g. by ESI–MS. Consequently, applications emerge for the PAS biopolymer itself, for example to accomplish chemical conjugation with synthetic peptides or small molecule drugs—which cannot be produced as fusion proteins using recombinant DNA technology—or as rheological additives in "biological" cosmetics and innovative food products.

However, to provide supply for such technical areas a highly efficient expression system as well as downstream process is needed that offers the potential to yield bulk quantities at an affordable price. Ideally, the bioprocess should avoid both cell harvest and disruption procedures as well as proteolytic cleavage processes in vitro. In this context, *Corynebacterium glutamicum*, a non-pathogenic Gram-positive bacterium [25] which is generally recognized as safe (known as GRAS status by regulatory authorities), offers the potential to produce PAS in high yields by way of ribosomal biosynthesis and to secrete this biopolymer, in a finally processed state, at high concentration into the culture medium, from which it may be recovered in a straightforward manner.

*C. glutamicum* was discovered as a fast-growing soil bacterium in 1957 in Japan and, since then, has gained tremendous biotechnological use for the industrial production of glutamate as a food ingredient. More recently, *C. glutamicum* provided the basis for the Corynex^®^ protein expression technology [26], which greatly simplifies the purification process of complex recombinant proteins and improves the biopharmaceutical drug development process by reducing production costs and time to market. We now report the high level production of long, monodisperse recombinant PAS biopolymers in *C. glutamicum*, their efficient purification and detailed biochemical analysis.

## Materials and methods

### Construction of plasmids for PAS biopolymer secretion

A DNA fragment containing a promoter of the *csp*B gene of *C. glutamicum* ATCC 13,869 and encoding a CspA signal peptide was amplified from pPSPTG1 template DNA (Table 1) using the forward primer 5′-ACGAATTCGAGCTCGGTACCCAAATTCCTGTGAAG-3′ and the backward/reverse primer 5′-CGACTCTAGAGGATCCGCTCTTCAGGCTGCCGTTGCCACAGGTGCGGC-3′. The forward primer contained a *Kpn*I recognition sequence and the reverse primer encoded an Ala codon followed by stop codon (both on the sense strand) as well as a *Sap*I and a *Bam*HI recognition sequence. The amplified DNA fragment was inserted into the *Kpn*I-*Bam*HI restriction site of pPK4 (Table 1) using the In-Fusion HD cloning kit (Takara Bio, Kusatsu, Japan) to generate the plasmid pPK4-PcspB-CspA-A-SapI. Correct insertion was confirmed by DNA sequencing on a 3500xL instrument (Applied Biosystems/Thermo Fisher Scientific, Waltham, MA, USA) using the BigDye terminator v3.1 cycle sequencing kit. In a similar manner, the plasmid pK4-PcspB-CspA-QSPA-SapI was constructed; however, in this case the reverse primer 5′-CGACTCTAGAGGATCCGCTCTTCAGGCAGGGCTCTGTGCCGTTGCCACAGGTGCGGC-3′ was used, where the Ala codon was preceded by nine nucleotides encoding Gln-Ser-Pro.

**Table 1:** Bacterial strains and plasmids used in this study

	Characteristics	References
*C. glutamicum* strains		
YDK010	ATCC13869 N-methyl-N-nitro-N-nitrosoguanidine mutant	[28]
YDK0107	YDK010::phoS(W302C)	[29]
YDK0107∆*pmt﻿*1	∆*pmt*1 mutant of YDK0107	This study
Plasmids		
pPK4	*C. glutamicum* / *E. coli* shuttle vector, Km^r^	[28]
pPSPTG1	pPK4 harbouring pro-MTG fusion gene with CspA signal peptide from *C. ammoniagenes*	[28]
pXL1-PAS#1(600)	pXL1 harbouring the PAS#1(600) gene cassette	[27]
pPK4-PcspB-CspA-A-SapI	pPK4 harbouring *csp*B promoter and encoding a CspA signal peptide, Ala and a *Sap*I site	This study
pPK4-PcspB-CspA-QSPA-SapI	pPK4 harbouring *csp*B promoter and encoding a CspA signal peptide, QSPA and a *Sap*I site	This study
pPK4-PAS#1(600)	pPK4 harbouring cspB promoter and encoding a CspA signal peptide fused with the PAS#1(600) biopolymer	This study
pPK4-QSP-PAS#1(1200)	pPK4 harbouring cspB promoter and encoding a CspA signal peptide followed by QSP and fused with the PAS#1(600) biopolymer	This study

A PAS gene cassette was designed for expression in *C. glutamicum* using a proprietary algorithm [27], resulting in a DNA sequence with low repetitiveness, minimal RNA secondary structure and optimized codon usage as well as G/C nucleotide content. Shorter building blocks of these complex gene sequences were individually synthesized and then assembled stepwise on a donor plasmid, pXL1 (Table 1), leading to a PAS#1(600) gene cassette encoding 600 Pro/Ala/Ser residues. On this cloning vector, the PAS#1(600) gene cassette is flanked by two *Sap*I restriction sites, whose type IIS recognition sequences are oriented in opposite directions and, upon digest, generate three-nucleotide complementary 5'-sticky ends that both encode an Ala residue (thus, actually generating a PAS reading frame comprising 601 residues in total). Finally, pXL1-PAS#1(600) was digested with *Sap*I to liberate the PAS#1(600) gene fragment, which was inserted into the singular *Sap*I site of pPK4-PcspB-CspA-A-SapI or of pPK4-PcspB-CspA-QSPA-SapI using the Mighty Mix DNA ligation kit (Takara Bio) to obtain the expression plasmids pPK4-PAS#1(600) and pPK4-QSP-PAS#1(1200), respectively—the latter harbouring the PAS#1(600) gene fragment inserted (by chance) twice in tandem.

### Construction of a *pmt*1 deletion mutant of *C. glutamicum*

A gene-disruption mutant of *C. glutamicum* was constructed according to a previously published procedure [26]. The plasmid pBS5T [30], which carries a temperature-sensitive replication origin and the *Bacillus subtilis sac*B gene, was used as a suicide vector [31]. First a 1.0-kb upstream region of the *pmt*1 gene was PCR-amplified from *C. glutamicum* ATCC 13,869 chromosomal template DNA using the primers 5′-GTTTCGTACCTAAACCGACCCGAACC-3′ and 5′-TCGGTTGATTGTAGAGCCTTGGCG-3′, and a 1.0-kb downstream region of the *pmt*1 gene was separately PCR-amplified using primers 5′-GCCAAGGCTCTACAATCAACCGATGGCCGAACCAAGTGACCAATGC-3′ and 5′-AAGCTCCGGCATTGATTCCGTTACC-3′. In the second step, the two PCR products were assembled in a new PCR, via a 23 bp overlap, using the flanking primers 5′-GTTTCGTACCTAAACCGACCCGAACC-3′ and 5′-AAGCTCCGGCATTGATTCCGTTACC-3′. The amplified DNA fragment was inserted into the *Sma*I site of pBS5T to obtain pBS5T∆pmt1. *C. glutamicum* YDK0107 was transformed with pBS5T∆pmt1 by electroporation, and kanamycin-resistant colonies were selected on CM2G agar plates at 34 °C. Because pBS5T does not replicate at 34 °C, only single-crossover chromosomal-integration strains could grow at 34 °C. One of the resulting kanamycin-resistant transformants was then propagated in CM2G liquid medium without kanamycin overnight, and the cells were finally spread on CM2G agar plates containing 10% w/v sucrose. Cells harbouring the *sac*B gene cannot grow in the presence of sucrose and, thus, only cells in which the *sac*B gene was excised from the chromosome by a second homologous recombination event could propagate on these plates. The resulting sucrose-resistant colonies either corresponded to the wild-type strain or had the *pmt*1 gene deleted, depending on the recombination points. The desired *pmt*1 deletion strain was finally selected by analytical PCR from chromosomal DNA of individual colonies using the same pair of flanking primers as described above and designated as YDK0107∆*pmt*1 (see Table 1).

### Fermenter production of the PAS biopolymers

The bacterial strains and plasmids used in this study are listed in Table 1. For the production of PAS#1(600) and Pca-PAS#1(1200), the protein-*O*-mannosyl transferase-deficient *C. glutamicum* strain YDK0107∆*pmt*1 was transformed with the expression plasmids pPK4-PAS#1(600) or pPK4-QSP-PAS#1(1200), respectively, via electroporation using a Gene Pulser (Bio-Rad, Hercules, CA, USA) according to the manufacturer’s protocol. Glycerol stocks were prepared from single colonies. Cultivation was carried out in a 1 L fermenter with 300 mL working capacity. 50 mL CM2G medium [28] supplemented with 25 mg/L kanamycin in a 500 mL Sakaguchi flask was inoculated with 50 µL of the glycerol stock and incubated under agitation for 24 h at 30 °C as pre-culture. 15 mL of this pre-culture was used to inoculate the fermenter (Biott Corporation, Tokyo, Japan) containing 300 mL MMTG-J medium [28] supplemented with 25 mg/L kanamycin [32]. The fermenter was operated at a temperature of 30 °C, corresponding to the optimal growth temperature of *C. glutamicum* [25], using an agitation rate of 650 rpm with an aeration of ½ V/V/min. The pH was automatically maintained at either pH 6.5 or pH 7.0 by the addition of ammonia gas via the airflow. After glucose consumption and at an optical density (OD) of ~ 120 (measured for a 1:100 dilution at 610 nm), which was reached at a cultivation time of approximately 24 h, the fermentation process was terminated. The culture supernatant was cleared from cells by filtration through an SLGV033RS Hydrophilic PVDF Millex-GV Filter Unit (Merck Millipore, Cork, Ireland) and the cell-free broth was stored at − 80 °C.

### Purification of the PAS biopolymers

After thawing, 40 ml of the cell-free culture supernatant was mixed with crystalline citric acid to a final concentration of 100 mM and the PAS polypeptide was precipitated by addition of ammonium sulfate to 25% saturation (1.0 M) at 20 °C under stirring. After 30 min, the precipitate was collected by centrifugation and resolubilized in CEX buffer (20 mM acetic acid/NH_3_ pH 4.5).

Residual ammonium sulfate was removed by dialysis against CEX buffer, and the protein solution was subjected to subtractive-mode cation exchange chromatography (CEX) on a Fractogel EMD SO_3_^−^ HiCap (M) column (Merck, Darmstadt, Germany). The flow-through was collected, followed by dialysis against 20 mM NH_3_/acetic acid pH 9.5, and subjected to subtractive-mode anion exchange chromatography (AEX) on a Fractogel EMD TMAE HiCap (M) column (Merck). After thorough dialysis against ultra-pure water, the pure PAS polypeptide solution was passed through a 0.2 µm Millex-GV syringe filter and finally lyophilized.

To accomplish complete cyclization of the N-terminal Gln residue of the PAS#1(1200) biopolymer, the dry substance was dissolved in 99% acetic acid and incubated at 50 °C for 4 h. After precipitation with diethylether, the PAS biopolymer was dried in a RVC 2–18 CDplus rotational vacuum concentrator (Martin Christ, Osterode, Germany) and dissolved in water.

### Biochemical characterization of the PAS biopolymers

SDS-PAGE was performed under reducing conditions using SurePAGE Bis–Tris 4–12% acrylamide gradient gels (Genscript, Piscataway, NJ, USA) with the corresponding Tris-MOPS-SDS buffer. For visualization of the PAS biopolymer, the SDS-Gel was subjected to a BaI_2_ staining procedure as described [9]. Briefly, the gel was rinsed with deionized water, incubated in a 2.5% (w/v) BaI_2_ solution for 10 min, rinsed with deionized water, and incubated in Lugol’s iodine (5% (w/v) I_2_, 10% (w/v) KI) for 10 min. The background was destained by incubation in deionized water with repeated water exchanges until an optimal contrast was achieved (5–10 min). The gel was then immediately recorded on a Perfection V800 document scanner (Epson, Meerbusch, Germany). Alternatively, the gels were stained with Coomassie brilliant blue R-250 using standard procedures. Western blots were performed as described previously [33]. Densitometric analysis of the scanned digital images was accomplished using the CLIQS software (TotalLab, Newcastle-Upon-Tyne, UK).

Size exclusion chromatography (SEC) was performed on a Superose 6 Increase 10/300 GL column (Cytiva, Uppsala, Sweden) in phosphate-buffered saline (PBS, Cat. No. 21-040-CV; Corning, Manassas, VA, USA). ESI–MS measurements were carried out on a maXis quadrupole time-of-flight (Q-TOF) mass spectrometer equipped with an electrospray ionization (ESI) source (Bruker Daltonics, Bremen, Germany) using the positive ion mode. Deconvolution of the raw spectra was performed via the Bruker Compass Data Analysis Software (ver. 4.3) by applying the MaxEnt or MaxEntX algorithms [34].

## Results and discussion

### PAS gene design and expression vector construction

The goal of this study was to secrete PAS biopolymers at two different lengths, comprising 600 and 1200 amino acid residues, respectively, into the culture medium of *C. glutamicum*. Our first strategy was simply based on the N-terminal fusion with a signal peptide, both directing secretion of the gene product out of the bacterial cell and ensuring accurate liberation of the first amino acid residue of the mature PAS moiety (e.g. Ala) via processing by the signal peptidase. Such PAS polypeptides are fully devoid of charged side chains. Hence, the only strongly polar and, potentially, chemically reactive groups occur at the amino- and the carboxy-terminus (${\text{NH}}_{3}^{ + }$ and COO^−^, respectively, at neutral pH).

The C-terminal carboxylate group offers site-specific chemical activation via ester chemistry in case conjugation with other functional molecules or materials is desired. However, under such circumstances the N-terminal nucleophilic amino group would lead to interference and should ideally be blocked. This can be implemented in the biotechnological production strategy without much effort at the genetic level by introducing a single Gln residue at the N-terminal position, directly downstream of the signal sequence. After proteolytic processing of the signal peptide in vivo, the emerging N-terminal Gln can cyclize spontaneously—or under enforced conditions by acidic treatment in vitro—to form a pyroglutamic acid (2-pyrrolidone-5-carboxylic acid, PCA) residue [35], which no longer acts as a nucleophile.

To investigate both production strategies, different PAS gene cassettes were designed. The PAS polypeptide sequence used in this study is based on consecutive 20mer amino acid repeats comprising Pro, Ala and Ser residues in a defined order to effectively prevent the formation of secondary structures [8]. The resulting random coil polypeptide adopts an expanded hydrodynamic volume as evident from size exclusion chromatography (SEC, see below) whereas the repeated sequence at the amino acid level guarantees uniform biophysical behaviour along the entire polypeptide chain [9].

To allow efficient biosynthesis in *C. glutamicum*, the encoding PAS gene cassettes were designed using a proprietary algorithm [27]. First, the codon usage was adapted for high expression in *C. glutamicum*, including optimization of the GC content and minimization of RNA secondary structures. Second, the sequence repetitivity at the nucleotide level was minimized—while precisely maintaining the repeated amino acid sequence—in order to suppress homologous recombination and to ensure high genetic stability during multiple replication cycles, in line with regulatory requirements for good manufacturing practice (GMP) [7]. In this manner, a synthetic non-repetitive PAS gene cassette encoding 600 amino acids (plus an additional N-terminal Ala residue for purposes of gene cloning, cf. Materials and Methods) was subcloned on the vector pXL-1 to yield pXL1-PAS#1(600) [27].

**Fig. 1:**
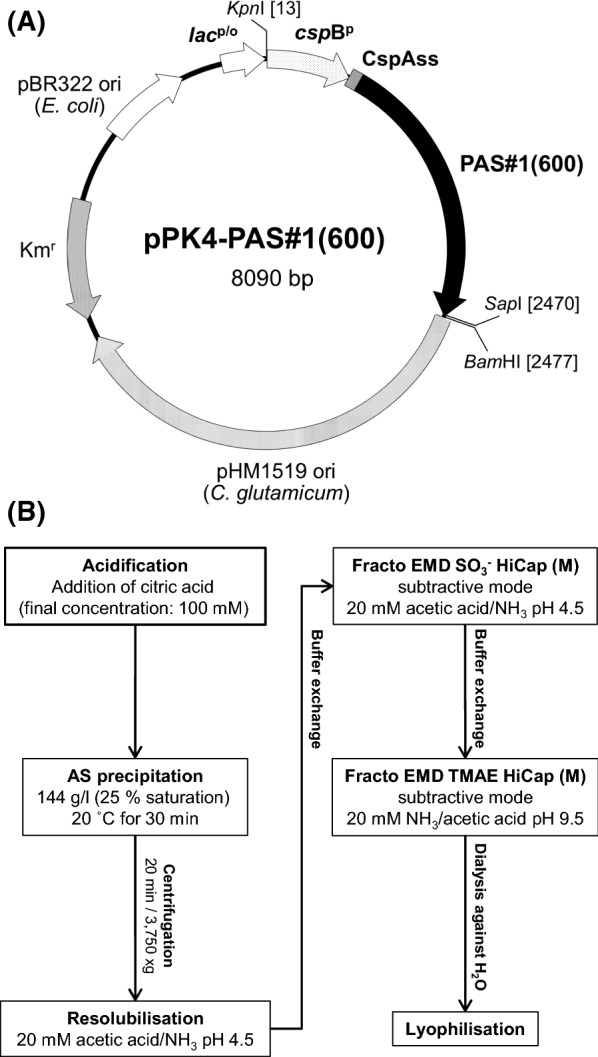
*C. glutamicum* expression vector for the secretion of PAS polypeptides (**A**) and schematic downstream process for the purification of the PAS biopolymers (**B**)

**Fig. 2:**
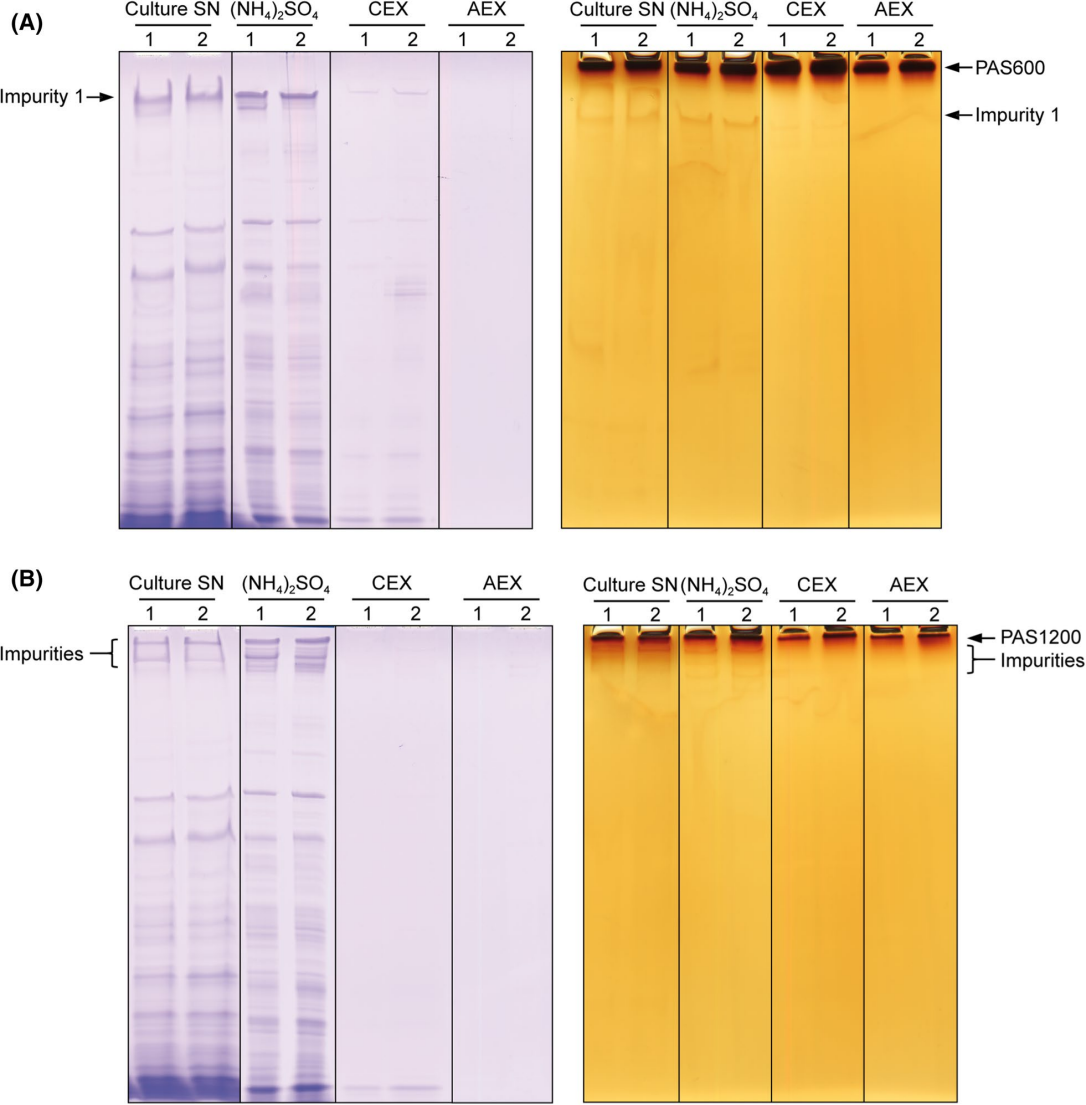
Analysis of PAS polypeptide secretion in *C. glutamicum* and of the downstream purification by SDS-PAGE. PAS#1(600) (**A**) and PAS#1(1200) (**B**) were produced in MMTG-J medium containing 25 mg/L kanamycin either at pH 6.5 (lanes 1) or at pH 7.0 (lanes 2). Samples from the culture supernatant (SN), the ammonium sulfate precipitate ((NH_4_)_2_SO_4_) and flow-through fractions from CEX and AEX were subjected to SDS-PAGE. The gels were either stained with Coomassie brilliant blue (left) or with BaI_2_ (right). Free PAS polymer is only visible with BaI_2_, not with Coomassie staining

For the production of PAS polypeptides in *C. glutamicum*, the following two expression plasmids were constructed (Table 1; Fig. 1): (i) pPK4-PAS#1(600), by insertion of one PAS#1(600) gene cassette into pPK4-PcspB-CspA-A-SapI via the singular *Sap*I restriction site; (ii) pPK4-QSP-PAS#1(1200), by insertion of two PAS#1(600) gene cassettes, in tandem, into pPK4-PcspB-CspA-QSPA-SapI. Both plasmids led to stable and efficient expression of the corresponding PAS polypeptide as will be described in the following (Table 2; Fig. 2).

**Table 2:** Characteristics and production parameters of PAS biopolymers

Name	Mature amino acid sequence	Mol. mass	Culture pH	Final yield (g/l)	Yield of purification (%)
PAS#1(600)	Ala-(SPAAPAPASPAAPAPSAPAA)_30_	49,644.3	6.5	3.6	98
			7.0	4.4	90
PAS#1(1200)	QSPA-(SPAAPAPASPAAPAPSAPAA)_60_	99,511.8	6.5	2.1	97
			7.0	3.1	94

### Cultivation of *C. glutamicum* for high yield PAS secretion

Employing the two expression plasmids, the following PAS polypeptides were produced in *C. glutamicum*, differing in length and N-terminal sequence, after processing of the signal peptide: (i) The PAS#1(600) polypeptide with a molecular mass of ~ 50 kDa and an N-terminal Ala residue with a free amino group, suitable e.g. for conjugation with other molecules or surfaces that carry activated ester groups; (2) the ultra-long PAS#1(1200) polypeptide with a mass of ~ 100 kDa and an N-terminal Gln residue (followed by Ser-Pro-Ala-[PAS]_1200_), which can be post-translationally converted into a chemically inert pyroglutamyl group such that only its C-terminal carboxylate remains available for chemical conjugation (Table 2). Both PAS sequences were N-terminally fused with the CspA signal sequence (CspAss) of *C. ammoniagenes* [26, 28] to facilitate Sec-dependent secretion into the culture medium. Since the addition of the Gln codon upstream of the N-terminal Ala residue as encoded on the standard PAS gene cassette would have created an ambiguous double recognition site for the bacterial signal peptidase I, thus causing imprecise cleavage of the signal sequence [36], the N-terminus was extended by the sequence Gln-Ser-Pro, which resulted in uniform processing (see below).

Both CspAss-PAS precursor polypeptides were expressed under control of the constitutive *csp*B promoter of *C. glutamicum* [26, 28]. For each construct, a batch-fermentation was performed in 300 ml MMTG-J medium [32] supplemented with kanamycin under aeration in an automated fermenter vessel. After glucose consumption, at around 24 h cultivation period, the cell density in each fermenter had reached approximately OD_610_ = 120 and the cultures were harvested. The cleared supernatant was prepared by sterile filtration.

*C. glutamicum* is a neutralophilic microorganism that grows optimally at neutral to moderately alkaline conditions, even though it can tolerate pH fluctuations in the range of 6.0 to 9.0 [37]. The effects of the external pH on the bacterial metabolism include, among others, the proton motive force, the solubility/accessibility of trace elements, the membrane lipid composition, the presence/activity of chaperons as well as of ion channels in the cell membrane [38, 39], which all can have an impact on the expression, secretion and stability of a recombinant protein. Furthermore, the pH may affect the stability of the secreted recombinant protein within the culture supernatant. Due to the unpredictability of the culture pH on the yield of recombinant protein, it has proven beneficial to test at least two pH conditions to identify the best one for high target protein yield. Consequently, two fermentation runs were performed for each PAS polypeptide, either at pH 6.5 or pH 7.0.

Initial experiments indicated *O*-glycosylation at the Ser residues present in the PAS#1 sequence when using *C. glutamicum* strain YDK0107 [26] (data not shown). Hence, to avoid possible product inhomogeneity, we constructed a protein-*O*-mannosyl transferase-deficient (∆*pmt*1) *C. glutamicum* strain [40] based on YDK0107 (see “Materials and methods”), which was employed for subsequent fermentations as above.

### Purification and biochemical characterization of PAS biopolymers

PAS biopolymers exhibit distinctive biophysical properties, in particular a strongly hydrophilic nature, despite lack of charged side chains, as well as amenability to ammonium sulfate precipitation at low concentration. These features enabled separation of the recombinant polypeptides from the (few) host cell proteins present in the culture supernatant of *C. glutamicum* with a lean purification procedure and at high yield. In brief, after the addition of citric acid (100 mM)—to inhibit potentially contaminating proteases—the cleared cell-free culture supernatant was subjected to ammonium sulfate precipitation, followed (i) by resolubilization in a low ionic strength buffer at pH 4.5 and (ii) subtractive cation exchange chromatography (CEX) as well as (iii) a final subtractive anion exchange chromatography (AEX) at pH 9.5 (Fig. 1B).

The purification steps were documented by SDS-PAGE using two different staining procedures (Fig. 2). Due to the complete lack of hydrophobic as well as charged side chains, the plain PAS polypeptides do not bind Coomassie dyes and, thus, remain invisible in conventionally stained SDS-PAGE. On the other hand, PAS biopolymers can be stained with BaI_2_, a procedure that was initially developed for the visualization of PEG in protein gels [9, 41]. Indeed, both the PAS#1(600) and the Gln-PAS#1(1200) polypeptides gave rise to prominent bands in BaI_2_-stained SDS-PAGE, close to the top of the running gel, but remained invisible after Coomassie staining – in contrast to the host cell proteins in the culture supernatant (see Fig. 2). The slow migration of PAS biopolymers in the SDS-PAGE results from their poor propensity to interact with SDS, whose negatively charged head groups provide the driving force during electrophoresis [9]; hence, PAS polypeptides appear at a much larger apparent molecular size in SDS-PAGE in comparision with conventional standard proteins than would be expected from the number of their constituent amino acid residues.

Notably, apart from typical host cell proteins running at lower, normal molecular weights, this analysis revealed a faint band in the same size range as the PAS#1(600) polypeptide which was stained both with BaI_2_ and Coomassie (dubbed “impurity 1”). This indicates an uncleaved precursor that still carries the predominantly hydrophobic CspA signal peptide, which presumably can confer Coomassie binding. Densitometric analysis showed that impurity 1 constituted 1.4–2.0% of the total BaI_2_-stainable protein. Due to its higher pI (with one Arg and two Lys residues close to the N-terminus of the CspA signal peptide), this impurity was efficiently removed by subtractive CEX. After the subsequent AEX, no further impurities were detectable with both gel staining techniques.

Finally, the PAS biopolymer was thoroughly dialyzed against ultra-pure water and lyophilized. The gravimetrically determined extrapolated yield of the PAS#1(600) polymer was 3.6 g per 1 L culture supernatant from the fermentation at pH 6.5, and 4.5 g/L from the fermentation at pH 7.0. For Gln-PAS#1(1200), the yields where 1.2 g/L and 2.1 g/L for the pH 6.5 and pH 7.0 fermentations, respectively. Moreover, quantification of the initial PAS concentrations in the cell-free culture supernatants by densitometry of BaI_2_-stained SDS-PAGE gels allowed the assessment of purification-related losses for the different PAS biopolymers. According to this analysis, the overall yields were > 90% (Table 2).

**Fig. 3:**
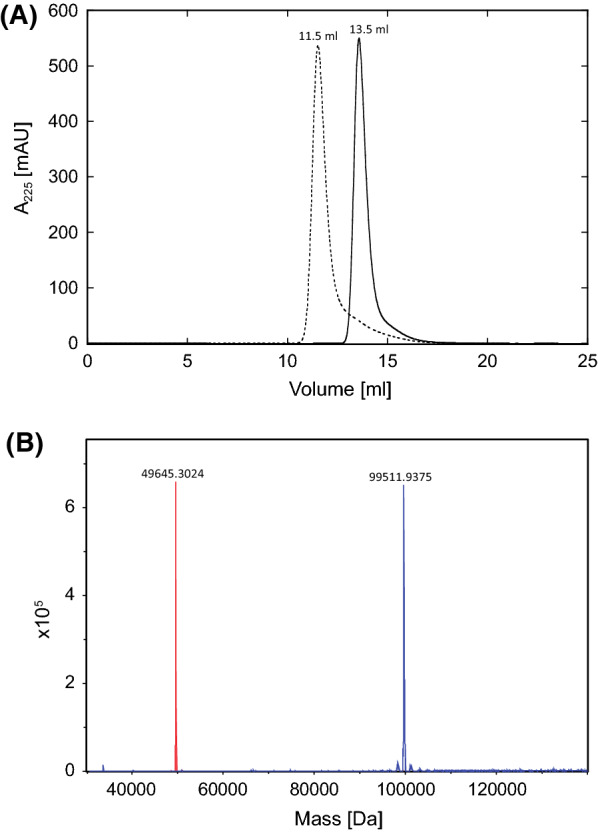
Biochemical analysis of the purified recombinant PAS biopolymers. **A** SEC profiles of PAS#1(1200) (dashed line) and PAS#1(600) (solid line). **B** Deconvoluted ESI–MS spectra (overlay) of PAS#1(600) (red, calculated mass: 49,644.3 Da) and PAS#1(1200) (blue, calculated mass with intact N-terminal Gln residue: 99,511.8 Da)

PAS biopolymers show very large hydrodynamic radii in SEC due to their dynamically unstructured nature [9]. When comparing the SEC elution volumes with a calibration curve of globular standard proteins, the hydrodynamic radius of the PAS#1(600) produced in *C. glutamicum* corresponded to an apparent size of 550 kDa while the PAS#1(1200) polypeptide even revealed an apparent molecular mass of 1.8 MDa (Fig. 3). Both biopolymers appeared uniform and showed only one sharp peak in the analytical SEC.

**Fig. 4:**
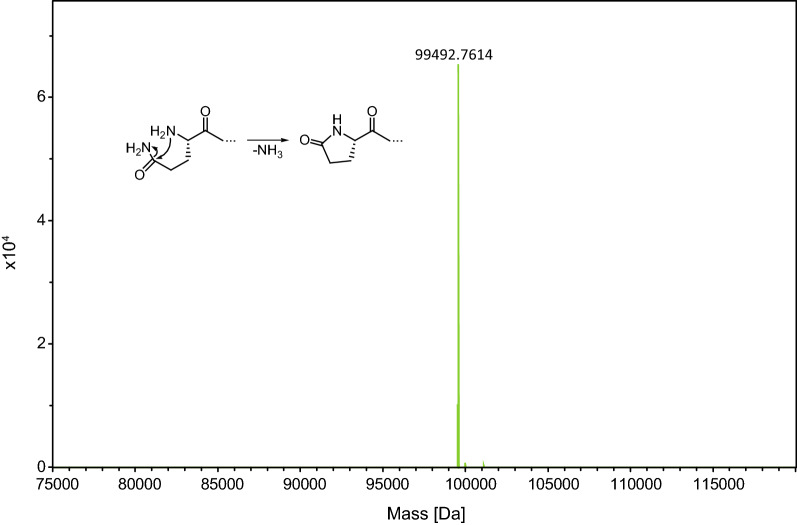
N-terminal pyroglutamate formation of the recombinant PAS#1(1200) biopolymer. The deconvoluted ESI–MS spectrum indicates complete cyclisation of the N-terminal Gln residue after incubation in 99% acetic acid at 50 °C for 4 h (calculated mass: 99,494.0 Da)

Furthermore, ESI–MS analyses confirmed the precise calculated masses (49,644.3 Da and 99,511.8 Da, respectively) for both the PAS#1(600) and the PAS#1(1200) polypeptides produced in *C. glutamicum* (Fig. 3B). No signs of inhomogeneity, potentially due to glycosylation, proteolysis, incorrect signal peptide processing or other posttranslational modification, were detectable. Interestingly, cyclization of the N-terminal Gln residue in the PAS#1(1200) polypeptide was neither triggered in vivo nor by the initial acidification of the culture supernatant prior to the purification process. However, after acetic acid treatment (99% v/v) of the purified biopolymer at 50 °C for 4 h, only the cyclized N-terminal pyroglutamyl group was detectable in ESI–MS (Fig. 4).

## Conclusions

We have established the efficient biosynthesis of a recombinant biodegradable PAS biopolymer using an established biotechnological production organism and Corynex® technology. Classical chemical synthesis of macromolecules by polymerization, as used for the production of PEG, generally yields a distribution of different lengths while so-called "monodisperse" polymer preparations require strong efforts for fractionation which is accompanied by significant losses [42]. In contrast, exploitation of the microbial protein synthesis machinery yields a uniform and perfectly defined PAS biopolymer, as evidenced by the single product peak in the ESI mass spectrum. The lean and efficient purification process described here is made possible (i) by the unique biophysical properties of PAS polypeptides, which distinguish them from any endogenous bacterial protein, (ii) the high secretion efficiency of the Corynex® expression system and (iii) the very low amount of contaminating host cell proteins found in the culture broth of *C. glutamicum*. As result, high yields of ≥ 2 g/L culture for the ultra-long PAS#1(1200) and even ≥ 4 g/L culture for the PAS#1(600) biopolymer were obtained after purification. It can be expected that strain optimization as well as fermentation and bioprocess development should further increase these yields, as demonstrated in other applications of the Corynex^®^ expression system before [26]. Consequently, the low cost of goods of the PAS biopolymer according to this novel production route offers the opportunity to compete with other polymers, such as PEG or polysaccharides, in market segments outside the pharmaceutical industry, for example, in the area of innovative cosmetics [43, 44].

Moreover, the biosynthesis of PAS polypeptides, which is based on a genetic blueprint, allows the simple site-directed functionalization with various reactive groups (e.g. thiol, amino or carboxyl groups) by placing appropriate amino acid residues in any desired position, thus enabling the preparation of multifunctional or branched PAS biopolymers for multivalent, bi- or multispecific drug conjugate formats. This versatility in combination with the high solubility in water as well as polar aprotic organic solvents [9], the lack of toxicity and immunogenicity [7, 8] and the accurately defined PAS chain length offer a broad range of applications compared to other hydrophilic polymers. In this context it is beneficial that the broad substrate tolerance of the signal peptidase I [36] that processes the signal peptide after secretion from *C. glutamicum* allows a variety of amino acids to be chosen for the N-terminus of the mature PAS biopolymer. Thus, we were able to encode a Gln residue at the N-terminus of the PAS#1(1200) polypeptide, which was easily converted into the chemically inert pyroglutamyl residue, thus leaving the carboxy-terminus as single reactive group for the site-specific coupling to various types of drug molecules using conventional peptide chemistry.

## Data Availability

All data generated or analyzed during this study are included in this published article.

## Abbreviations

AEX: Anion exchange chromatography
CEX: Cation exchange chromatography
CspAss: CspA signal sequence
PAS: Pro/Ala/Ser
PBS: Phosphate-buffered saline
